# Faster phonological processing and right occipito-temporal coupling in deaf adults signal poor cochlear implant outcome

**DOI:** 10.1038/ncomms14872

**Published:** 2017-03-28

**Authors:** Diane S. Lazard, Anne-Lise Giraud

**Affiliations:** 1 Institut Arthur Vernes, ENT Surgery, 36 rue d'Assas, Paris 75006, France; 2 Inserm U 1127, CNRS UMR 7225, Sorbonne Universités, UPMC Univ Paris 06 UMR S 1127, Institut du Cerveau et de la Moelle épinière, ICM, F-75013 Paris, France; 3 University of Nottingham, School of Medicine, University Park Nottingham NG7 2UH, Nottingham, UK; 4 Department of Neuroscience, University of Geneva, Campus Biotech, 9 chemin des Mines, Geneva 1202, Switzerland

## Abstract

The outcome of adult cochlear implantation is predicted positively by the involvement of visual cortex in speech processing, and negatively by the cross-modal recruitment of the right temporal cortex during and after deafness. How these two neurofunctional predictors concur to modulate cochlear implant (CI) performance remains unclear. In this fMRI study, we explore the joint involvement of occipital and right hemisphere regions in a visual-based phonological task in post-lingual deafness. Intriguingly, we show that some deaf subjects perform faster than controls. This behavioural effect is associated with reorganized connectivity across bilateral visual, right temporal and left inferior frontal cortices, but with poor CI outcome. Conversely, preserved normal-range reaction times are associated with left-lateralized phonological processing and good CI outcome. These results suggest that following deafness, involvement of visual cortex in the context of reorganized right-lateralized phonological processing compromises its availability for audio-visual synergy during adaptation to CI.

## Abstract (web summary)

Cochlear implants have variable outcomes for adult deafness. Here the authors show that fast responses and specific recruitment of right temporal cortex on a simple visual rhyming task strongly predicts poor implant performance.

Predicting individual benefit following a cochlear implant (CI) during post-lingual deafness remains a significant challenge. Altogether, clinical factors such as the duration and aetiology of deafness, age and amount of residual hearing do not appear to account for >20% of outcome variance^1^. An important source of variability presumably lies in the cognitive ability to make use of an implant, and in particular the capacity to compensate for the crude CI auditory input by mapping auditory and visual speech cues^2^,^3^,^4^. Accordingly, the response of visual cortex to speech, shortly after implantation, is a significant positive predictor of CI success in post-lingual deaf adults^4^,^5^,^6^. By contrast, abnormal functional activation of the right temporal cortex by visually presented linguistic inputs or even by basic visual stimuli during post-lingual deafness and after auditory recovery with a CI consistently appears to be a negative predictor of CI success^7^,^8^,^9^. Whether these two neurofunctional markers are independent or whether they jointly contribute to CI outcome is unknown.

When losing hearing, post-lingual deaf subjects try to rely on lip-reading to maintain oral communication. Surprisingly, however, lip-reading does not seem prone to improvement following deafness. It even seems to deteriorate over time^10^, presumably because it depends on multimodal circuits that are moderately plastic in adulthood^11^,^12^,^13^,^14^,^15^,^16^,^17^, and also because it is not reinforced by auditory feedback. After cochlear implantation however, when deaf adults can rely again on the auditory sense, lip-reading does not decline^2^. It even slightly improves thanks to restored audio-visual cooperation^5^,^6^. Individual differences in CI success may therefore depend not only on the ability to combine audio-visual information following CI, but also on lip-reading skill before implantation. A causal relationship between lip-reading fluency before implantation and speech comprehension after implantation seems intuitive, but has so far not been confirmed by clinical or research data in the post-lingual deaf population. This might be because the availability of visual cortex for audio-visual remapping after implantation is not only determined by lip-reading circuitry before and during deafness, but presumably also by another form of deafness-induced brain reorganization, which involves the abnormal recruitment of the right hemisphere in phonological processing, notably the right temporal cortex^8^,^9^,^18^,^19^.

Left-hemispheric dominance for language in the human brain permits optimal, rapid, intra-hemispheric interaction between the left inferior frontal speech production region (Broca's area) and the temporal and occipital regions receiving linguistic input^20^,^21^,^22^. Left dominance is so strongly rooted that it is also present in congenitally deaf people who use sign language^23^,^24^. It can be challenged, however, when there are structural and/or functional anomalies in left hemispheric language networks, such as in adult post-stroke aphasia, or in developmental stuttering or dyslexia^25^,^26^,^27^,^28^,^29^. In these pathologies, the right hemisphere can reorganize to take over the impaired function. This allows for some degree of functional compensation^30^, but often leads to maladaptive plasticity^28^,^29^. That the involvement of the right hemisphere in speech processing during post-lingual deafness has repeatedly been associated with poor CI proficiency^7^,^8^,^9^,^18^ denotes that its functional involvement is not optimal for this function, and also perhaps that this reorganization preempts the visual cortex and prevents it from playing its role during the recovery phase.

The aim of the present study is to assess the relationship between right temporal cortex reorganization during profound post-lingual deafness and the availability of visual cortex for speech recovery after cochlear implantation. We asked adults with acquired profound deafness, candidates for a cochlear implantation, and normal-hearing controls to perform a challenging visual-based phonological task (a rhyming task) involving pseudo-homophones^31^,^32^, while we measured neural responses with fMRI. This task was designed to probe neural reorganization underlying phonological processing following post-lingual deafness. We specifically examined individual differences in functional connectivity between regions activated by the task, and hypothesized that the interaction between visual cortices and right temporal cortex would predict poor CI-outcome, as assessed 6 months after surgery.

Intriguingly, some deaf people perform the phonological task faster than controls. These faster subjects show a joint involvement of the right temporal cortex and bilateral visual cortices, poor lip-reading skill before CI, and poor subsequent CI outcome. Conversely longer reaction times are associated with preserved left-hemispheric dominance for phonological processing, good lip-reading skill before CI, and good subsequent adaptation to CI (that is, good speech perception). These findings suggest that fast and accurate phonological processing may be a marker of right hemisphere reorganization for speech in adult deafness. Testing CI candidates with visually-based rhyming tasks preoperatively could assist in identifying patients at risk of becoming non-proficient CI users.

## Results

### Behaviour

We used behavioural measurements to explore phonological processing in 18 deaf adult CI candidates and 17 normal-hearing controls matched for age and educational level (Supplementary Table 1). With a difficult rhyme decision task involving pseudo-homophones presented on a screen, we explicitly probed vision-based phonology, that is, grapheme to phoneme conversion and mental manipulation of speech sounds usually involving the occipital cortex and the dorsal phonological pathway^33^,^34^. Pseudo-homophones are non-words that are pronounced like words. They require a detailed phonological analysis before accessing their lexical content. The task consisted in saying whether two pseudo-homophones presented on a screen rhymed. A control task, randomly presenting two pseudo-homophones (same display as that of the rhyming task), was based on word spelling; it controlled for reading, motor planning, and working memory effects. We collected accuracy and reaction times to explore two distinct aspects of phonological processing, reliability and access to phonological representations, respectively.

**Figure 1:**
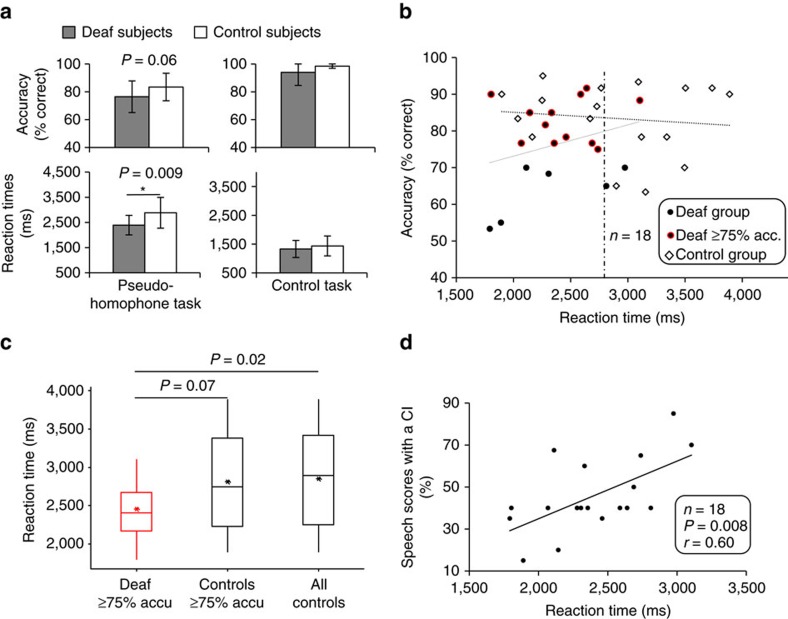
Behavioural results. (**a**) Mean performance and reaction times (% correct and RTs in milliseconds ms±s.d.) during phonological (rhyming decision on pseudo-homophones) and control (orthography decision) tasks in post-lingual deaf and normal-hearing control subjects. * Significant difference at *P*<0.05. (**b**) Phonological performance (% correct) as a function of RT (ms) in deaf (black dots with and without red-circle) and control (open diamonds) subjects. There was no statistical correlation between RT and performance in either group (the grey dotted lines represent theoretical linear regression lines). The deaf subjects who performed ≥75% correct are circled in red. The controls' mean and median RT is represented by a vertical dotted line (similar number: 2,728 and 2,757 ms, respectively). (**c**) Box plots of RTs in those deaf subjects who performed ≥75% correct (in red) and controls (good performers ≥75% and whole group). These good deaf performers had significantly faster RTs than the control group. Horizontal bars represent the medians and asterisks the means. (**d**) Post-cochlear implant (CI) scores (speech comprehension, % correctly repeated words) in post-lingual deaf subjects as a function of RT during the phonological task. A statistical correlation shows that the fast responders became poor proficient CI users.

The deaf subject group performed on average as accurately as, but significantly faster than, the control group (Fig. 1a: *T*-tests, *n*=18 and 17, *T*-value=−1.94, DF=33, *P*=0.06 (trend to perform less accurately), and *T*-value=−2.81, DF=33, *P*=0.009, respectively). Taking into consideration the trend of the deaf group to perform less accurately, we hypothesized that at the group level, deaf subjects could be faster but slightly less accurate than controls, possibly reflecting a difference in speed-accuracy trade-off. This effect was specific to phonological processing as there was no accuracy or reaction time (RT) difference between groups for the orthographic task.

To examine a possible speed-accuracy trade-off in deaf subjects, we looked at the relationship between RT and accuracy at the individual level (Fig. 1b). The absence of correlation and statistical relationship in either of the two groups (Pearson correlation, *n*=18 deaf: *P*=0.24, *r*=0.29, regression equation: (Acc=56.0+0.009 RT), *F*=1.49; *n*=17 controls: *P*=0.66, *r*=−0.12, regression equation: (Acc=88.8−0.002 RT), *F*=0.21) indicates that speed and accuracy were not directly related in our task and group samples. Critically, those deaf subjects who had good phonological performance (≥75%, black dots with red circles in Fig. 1b) performed significantly faster than controls (Fig. 1c: *T*-tests, *n*=12 and 17, *T*-value=−2.51, DF=26, *P*=0.02 for deaf≥75% versus all controls, and *T*-tests, *n*=12 and 14, *T*-value=−1.92, DF=20, *P*=0.07 for deaf≥75% accuracy versus controls≥75% accuracy). These behavioural data denote a substantial variability in the way some deaf subjects performed phonological operations. Importantly, no deaf subject presented with longer RTs than controls (eight controls had longer RTs than the ‘slowest' deaf subject, Fig. 1b).

We followed-up on our subjects after they received a CI and assessed their speech perception 6 months after implant surgery. We tested for a relationship between speech scores with the CI and behavioural measures before implantation, and found that RT during the phonological task, but not accuracy, significantly predicted speech perception after CI (RT in Fig. 1d: Pearson correlation, *n*=18, *P*=0.008, *r*=0.60; accuracy in Supplementary Fig. 1a: Pearson correlation, *n*=18, *P*=0.57, *r*=0.14. Multiple linear regression equation: (CI scores=−17.1+0.023 RT – 0.053 Accy) with *P*=0.012 for RT and *P*=0.90 for accuracy, *F*=4.32). This finding indicates that short RTs during the phonological task predicted poor CI speech scores, and vice versa.

Eleven out of the original 18 deaf patients agreed to take part in a functional magnetic resonance imaging (fMRI) study before cochlear implantation surgery. These subjects as well as 11 normal-hearing controls (out of the original 17 controls) matched with respect to age and educational level performed the phonological rhyming task in the scanner. We verified that the behavioural effects observed in the original samples of 18 deaf and 17 control subjects held in the sub-samples of 11 deaf and 11 control subjects. The 11 deaf subjects performed 400 ms faster on average, even though this difference was not significant in this smaller group (*T*-tests, *n*=11 in both groups, *T*-value=−1.62, DF=20, *P*=0.1). Phonological processing accuracy was similar between the two groups, with a trend toward better performance in controls (*T*-tests, *n*=11 in both groups, *T*-value=−2.01, DF=20, *P*=0.06, same result as in the whole group). Within the deaf group, the direction of the correlation between post-CI speech scores and RTs during the rhyming task (Supplementary Fig. 1b, black dots) was still positive (Pearson correlation, *n*=11, *P*=0.1, *r*=0.52).

In these same 11 deaf subjects, we also measured pre-implant lip-reading scores and tested for possible correlations with accuracy and RT on the phonological task. A significant positive relationship was obtained between lip-reading and RTs, with better lip-reading being related to slower RTs (Pearson correlation, *n*=11, *P*=0.03, *r*=0.64, linear regression equation: (Lipreading=−31.4+0.04 RT) with *F*=6.35; Supplementary Fig. 1b, yellow dots). Neither accuracy during the phonological task (black dots, Supplementary Fig. 1c), nor post-CI speech scores (red dots, Supplementary Fig. 1c) was related to lip-reading.

### Functional neuroimaging

**Figure 2:**
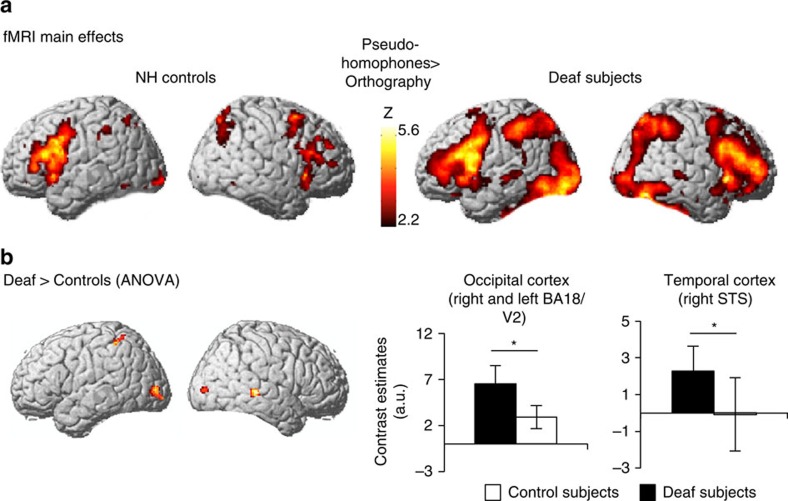
FMRI main effects. (**a**) Surface rendering of the main effects of phonological processing (*P*<0.05, FDR corrected for multiple comparisons) during the pseudo-homophone rhyming task in both groups (relative to the orthography control task). (**b**) Left: Surface rendering of the Deaf>Controls ANOVA results (*P*<0.05, FDR corrected for multiple comparisons). Right: parameter estimates in deaf subjects (black bars), and in normal hearing controls (white bars) in visual cortex (BA18/V2, left histogram, note that the pattern is similar in right and left visual cortex) and in the right posterior superior temporal sulcus (STS, right histogram). *indicates significant difference at *P*<0.05.

We performed three different analyses on the fMRI data. We first examined main effects of the rhyming task in each group, excluding potential effects of age. Normal-hearing controls activated bilateral inferior frontal and parietal cortices and the left ventro-lateral occipital cortex at a location compatible with the so-called visual word form area^35^ (*x y z* coordinates in MNI space=−54 −50 −18, Fig. 2a). Overall the same brain regions were activated in both groups, but neural activations were more pronounced and widespread in the deaf group, and there was additional activity in the bilateral temporal cortex. In a direct group comparison, activity in bilateral visual cortex, the right posterior superior temporal sulcus (STS) and the left superior parietal cortex (Supplementary Table 2) was enhanced in deaf subjects relative to controls (Fig. 2b left and 3 dark blue). In the visual cortex, activations were found for both groups, with over-activation in deaf compared to control participants (Fig. 2b, left histograms). In the right posterior STS, the activation observed in the deaf was at baseline in controls (Fig. 2b, right histograms). At the same statistical threshold, there was no region that controls activated more than deaf participants. These results concur with previous observations showing that visual cortex and right posterior temporal cortex undergo functional reorganization during deafness^6^,^9^,^18^,^36^,^37^, and confirm that the task probed critical deafness-induced reorganization.

**Figure 3:**
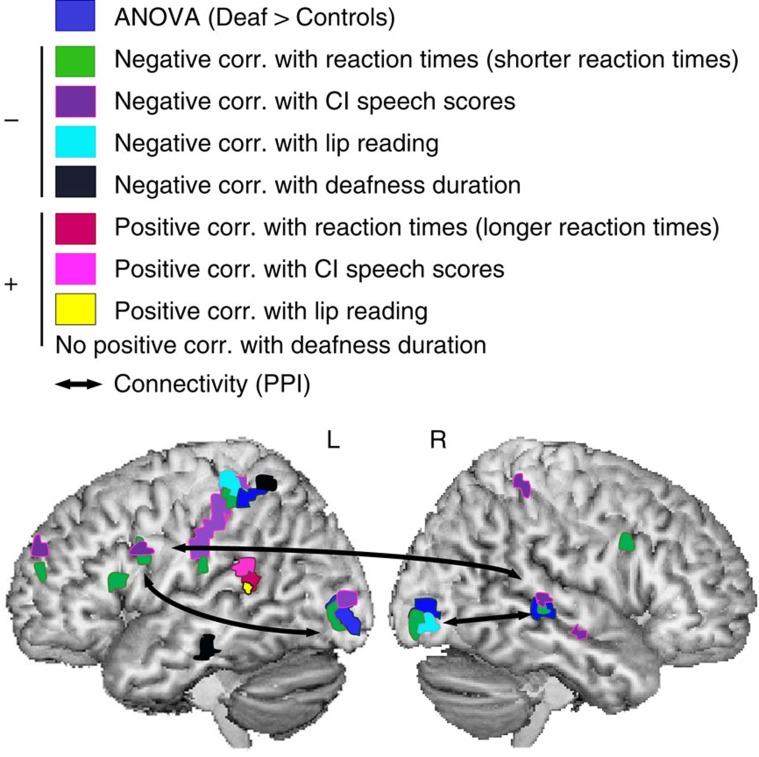
Summary of functional neuroimaging analyses. Group effects, correlations with behavioural and clinical variables, and functional connectivity in deaf subjects. All effects displayed on the figure are significant at *P*≤0.001, uncorrected. In dark blue: surface rendering of the ANOVA deaf subjects>controls (phonology>orthography); negative and positive correlations with RT during the phonological task are displayed in green and red, respectively; negative and positive correlations with CI scores are in purple and pink, respectively; negative and positive correlations with lip-reading scores measured before implantation are in cyan and yellow, respectively; negative correlation with deafness duration is displayed in black. Note that there was no controls>deaf subjects effect, and no positive correlation with deafness duration. Black arrows indicate significant connectivity between the seed regions (visual cortex and STS) and the inferior frontal gyrus in the phonological task.

Since behavioural results indicated a statistical relationship between RT and CI speech scores, and between RT and lip-reading (Fig. 1 and Supplementary Fig. 1), we explored how behaviour translated into neuro-functional patterns in the deaf group (Fig. 3) and how the latter predicted CI outcome. We assessed whether there was spatial overlap between the brain regions showing a functional association with RT in the phonological task and those associated with lip-reading and CI speech scores. We performed whole brain correlations with these clinical and behavioural measures, and kept all regressors separate (non-orthogonalized) in order to estimate both the common and shared variance, and in order to display spatial overlap across effects. Negative correlations with RTs (shorter RTs/stronger neural activity, shown in green in Fig. 3) were found at the exact location where deaf subjects presented stronger neural activations than controls: in bilateral visual cortex, the right posterior STS and the left superior parietal cortex/postcentral gyrus (Supplementary Table 3). This spatial congruence (green/dark blue overlap in Fig. 3) suggests that higher neural activation relative to controls contributed to faster responses in deaf subjects. There was also a negative relationship with RT (shorter RTs) in fronto-parietal regions (shown in green in Fig. 3). These effects were specific to the deaf group, and not related to motor control or task difficulty as they were controlled for by the orthographic task, and since there was no such activation in the control group (Supplementary Fig. 2). Negative regression with post-CI speech scores (Fig. 3, purple) showed a large overlap with the brain regions that correlated with fast responses (fronto-parietal regions, the left visual cortex and the right posterior STS), suggesting that the behavioural relationship between RT and post-CI scores might be underpinned by neural activity in these regions. We found further spatial overlap between the left superior parietal lobe/postcentral gyrus and the right visual cortex, that is, regions associated with fast phonological processing but poor CI outcome, and the regions that negatively correlated with lip-reading (Fig. 3, cyan).

The whole brain search for a positive correlation with RT (longer RTs, Fig. 3, red) revealed a single effect in the left posterior STS/superior temporal gyrus (STG). Interestingly, the positive correlations with CI speech scores and lip-reading converged onto this region (Fig. 3, pink and yellow, respectively), confirming the function of the left posterior STG/STS region as a critical ‘hub' for sensory-motor speech integration^38^,^39^,^40^,^41^. The overlap with the longer RT effect denotes that when audio-visual speech processes are left-lateralized despite profound hearing loss, RTs remain within normal-range (Fig. 1b).

To assess the dynamics of the above-reported effects, we finally explored the neural effects of duration of deafness, defined as severe-to-profound hearing loss, using a whole brain regression analysis. There was no positive effect of deafness duration, but a negative one was observed in the left superior parietal lobe and in the left inferior temporal gyrus (Fig. 3, black).

In summary, bilateral visual cortex, left superior parietal lobe and right posterior STS were over-activated in deaf subjects relative to controls. This enhanced neural activity was related to faster phonological processing, poor lip-reading ability, and poor CI speech perception. In addition, faster phonological processing and poor CI outcome were associated with enhanced neural activity in the left fronto-parietal region. Conversely, the activation of the left posterior temporal cortex was associated with the preservation of normal speed in phonological responses and with proficient lip-reading, and predicted good CI outcome. These results were not explained by an effect of deafness duration.

### Psychophysiological interactions

The goal of the next analysis was to test how the cortical regions revealed by the deaf>controls contrast interacted with each other and with the rest of the brain. Based on our hypothesis that a functional connection between visual cortex and right temporal cortex might modulate CI outcome, we explored functional connectivity of the visual cortex (Brodmann area 18) and the right posterior STS (seed regions) using psychophysiological interactions (PPI)^42^. In controls, the left visual cortex appeared to be functionally coupled with the left superior parietal lobe and the visual word form area^35^ (Supplementary Fig. 3, shown in green and yellow). As expected from the results of Fig. 2b (right histogram), the right posterior STS was not functionally connected with other brain regions when controls performed the phonological task. In deaf subjects, however, both the left visual cortex and the right posterior STS were functionally coupled with the left inferior frontal gyrus. This enhanced connectivity effect spatially overlapped with response speed and CI-score related effects in the left inferior frontal gyrus (Fig. 3, arrows, and Supplementary Fig. 3 in blue/red).

**Figure 4:**
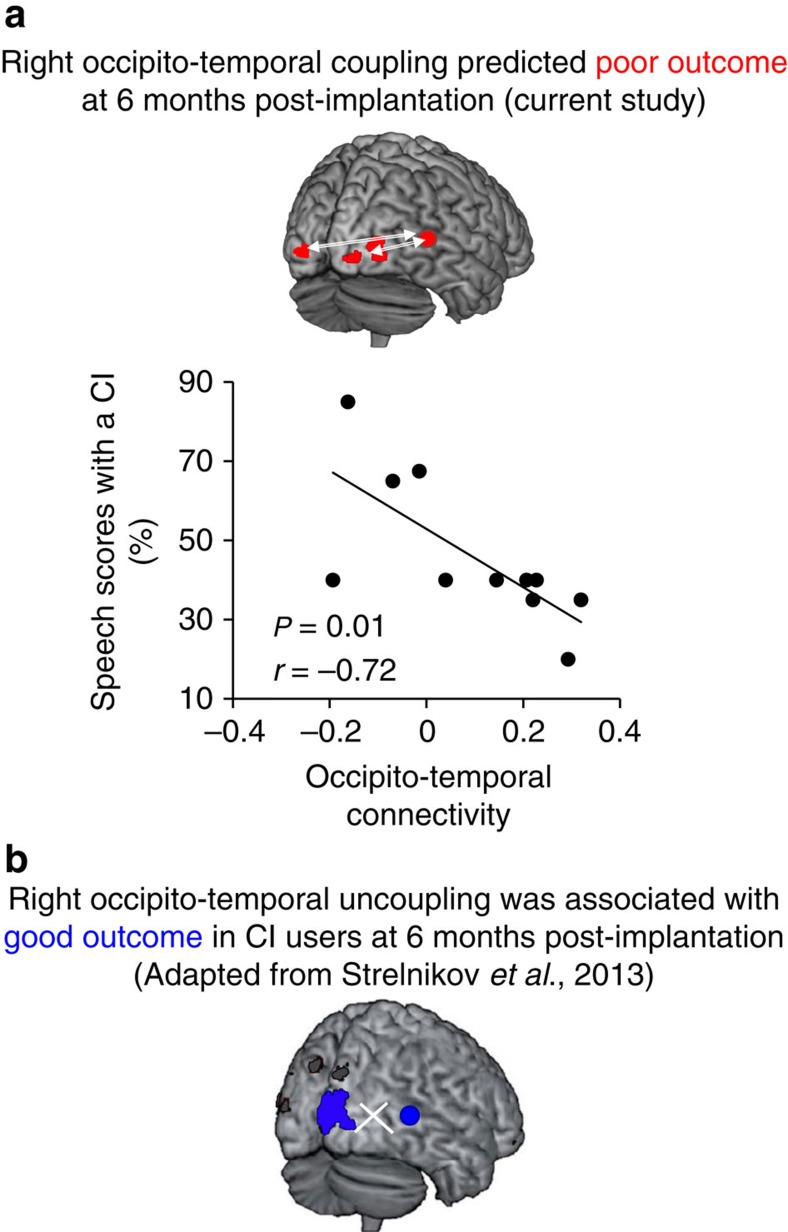
Right occipito-temporal coupling predicts occipital cortex availability for audio-visual synergy after auditory rehabilitation. (**a**) Surface rendering and plot of the negative correlation (in red) between individual strength of occipito-temporal coupling (current study: interaction term of PPI analysis between the right STS (seed region) and the right and left visual cortices (arrows)) and speech perception scores obtained 6 months after cochlear implantation (CI). The plot illustrates that the functional coupling between the left and right visual cortex and the right temporal cortex during deafness is a negative predictor of future CI proficiency. For illustration purposes, the extracted values from the coupling with the left occipital cortex only are displayed. Similar significant statistics were obtained for the right occipital cortex. (**b**) Surface rendering adapted from Strelnikov *et al*.^6^ showing that the absence of right occipito-temporal interaction during an audio-visual task in CI users 6 months after surgery is related to good speech perception scores (in blue).

The critical result of this analysis is that the right posterior STS interacted with bilateral visual cortex during the phonological task in deaf subjects. We hence verified the relevance of this functional coupling in CI outcome, and found that connectivity strength between the right posterior STS and the visual cortices (the PPI interaction terms for each occipital area independently) during the phonological task significantly predicted poor CI scores (Fig. 4a).

## Discussion

We hypothesized that the way the language system rewires during adulthood in acquired post-lingual deafness, under the dual pressures of audio-visual and writing-based communication strategies, has important consequences for subsequent CI outcome. On the one hand, auditory recovery with a CI relies on the availability of the visual cortex in the post-implantation phase, because the latter permits the remapping between visemes (lip movements) and perceived phonemes^5^,^6^,^7^. Auditory input from the CI is crude^43^ and patients must learn the fine correspondence between these new acoustic patterns and previously learned speech sound representations^18^,^44^. On the other hand, enhanced involvement of the right temporal lobe in speech processing, which occurs during deafness^9^,^18^ and persists after implantation^6^,^7^,^8^, seems detrimental to auditory recovery. We thus decided to explore how post-lingual deaf subjects deal with difficult rhyming decisions that require converting orthographic to phonological representations (memorized speech sounds^32^,^33^). Our aim was to address how the concurrent implication of visual cortices and right temporal cortex in phonological processing during post-lingual deafness might interfere with adaptation to CI.

When losing hearing, post-lingual deaf individuals progressively rely on vision to communicate^2^. Because their auditory function was normal or quasi-normal during childhood, they do not learn sign language, and lip-reading progressively becomes an essential means of communication as hearing deteriorates. Yet, lip-reading capacity is moderately plastic in adulthood^16^,^17^ and does not improve much in people who are not already proficient at the onset of deafness^11^. The absence of a positive effect of deafness duration on neural responses in the phonological task in the present study, in particular in the left STS/STG, where audio and visual speech stimuli are combined^45^, is in agreement with the sparse previous findings suggesting limited plasticity for lip-reading in deaf adults^16^ Considering this limited plasticity, investing in writing-based exchanges might be a faster and more rewarding communication strategy for some deaf adults than lip-reading. Accordingly, clinical observations indicate that post-lingual deaf subjects also turn to writing and reading to maintain social interactions.

A striking finding of the present study was that post-lingual profoundly deaf subjects on average performed the difficult phonological (rhyming) task more rapidly than normal-hearing controls. Although the deaf subjects tended to exhibit reduced accuracy, reflecting the fact that memory for speech sounds degrades when not maintained by accurate auditory input^9^,^18^, the effect was not explained by a simple speed-accuracy trade-off (Fig. 1b, dotted lines). In addition, 12 out of 18 deaf subjects showed fast but accurate performance (faster RTs than the controls, and accuracy≥75%, Fig. 1c). Importantly, in the deaf subjects, RT but not accuracy predicted speech comprehension ability six months after implantation: faster performance predicted worse post-CI speech scores (Fig. 1d).

Neurally, our deaf participants engaged the dorsal phonological route, as hearing subjects normally do^46^,^47^,^48^, when performing a phonological task. They additionally engaged bilateral visual cortex, the right posterior STS and the left superior parietal cortex. Activity in the latter region decreased in relation to deafness duration (Fig. 3, black). The left superior parietal cortex is typically involved in functions including auditory memory retrieval^49^,^50^; it is thus likely that the reduced involvement of this region in post-lingual deaf subjects reflects a deterioration of auditory memory retrieval in the absence of accurate auditory input^9^. Importantly, greater activation in the same four regions; that is, bilateral visual cortex, the right posterior STS and the left superior parietal cortex, as well as the opercular and triangular parts of the left inferior frontal gyrus (Broca's area^32^,^38^), was associated with faster RTs in deaf subjects (but not in controls, Supplementary Fig. 2) during the phonological task.

A key finding of the study was that the right temporal cortex was both abnormally involved in phonological processing in deaf individuals, and associated with faster responses and poor CI outcome. The absence of activation in right posterior STG and STS in controls (Fig. 2a,b) confirmed that these regions do not primarily contribute to phonological processing^38^,^39^. The right STG is normally involved in the analysis of paralinguistic speech cues, prosody and environmental sounds^51^,^52^,^53^,^54^, while the right STS processes biological motion^55^,^56^, such as face and eye movements^40^, and facial emotional expressions^57^. The right posterior STS integrates auditory and visual input and is therefore typically involved in voice-face associations^58^,^59^. Its recruitment in deaf subjects in the present study may indicate a change of function. Similar to a previous study where in post-lingual deaf adults the functional specialization of the right temporal lobe (STG) shifted from environmental sound to phonological processing^9^, the current results could reflect a deafness-related shift from voice/facial emotion recognition to written speech processing within the right STS. This hypothesis concurs with recent findings in deaf children^60^ suggesting that CI users are impaired in facial expression processing. Altogether, the current findings confirm that the right STG/STS region undergoes functional reorganization in post-lingual deaf subjects (see ref. 61 for a review). By analogy with the recruitment of specialized modules of the occipital cortex for different linguistic functions in blind subjects^62^, discrete sub-regions of the right temporal cortex might have become reorganized for specific aspects of speech processing.

Our specific hypothesis was that CI outcome depends on the availability of the visual cortex for audio-visual remapping in relation with left-lateralized phonological processing during auditory rehabilitation, and should therefore be compromised if visual cortex participates in a reorganized phonological circuit during deafness. Functional connectivity analyses showed that right and left visual cortices jointly participated in an abnormal right hemispheric phonological network, which predicted poor CI outcome (Fig. 4a). Both regions interacted directly with the left inferior frontal gyrus, by-passing key steps observed in controls, ones involving the visual word form area and the left superior parietal lobe (Supplementary Fig. 3). Thus, faster RTs might be explained by a more direct access to high-order phonological areas in deaf subjects (Fig. 3, black arrows, and Supplementary Fig. 3), which may counteract the fact that the recruitment of the right hemisphere should in principle slow down phonological processing. Similar to what has been observed in sight-deprived subjects, right temporal cortex reorganization and the reinforcement of its connectivity with visual areas could be facilitated by a top-down effect from the frontal lobe^62^. Crucially, neural activity in the visual cortex during visual-based phonology was related to poor lip-reading ability (Fig. 3, cyan), and neural activity in both the visual cortex and the right STS predicted poor CI performance (Fig. 3, purple). In addition, the connectivity strength between the right STS and bilateral visual cortex predicted poor CI speech perception outcome (Fig. 4a). These results confirm our hypothesis that reorganization of the right temporal cortex is not independent from that of visual cortex, and that they likely conjointly determine CI outcome.

The results also emphasize that maintaining language processing within the left hemisphere was associated with good CI outcome. We found a positive correlation between RT and lip-reading ability (Supplementary Fig. 1b). Subjects with better lip-reading scores had longer RTs, and because longer RTs in deaf people were within normal range (Fig. 1b), this could suggest that having efficient lip-reading prevents maladaptive phonological reorganization within the right temporal lobe and ensures good CI performance. Accordingly, we observed that those deaf subjects who had good pre-implant lip-reading scores used the left posterior STG/STS region to perform the phonological task, and later displayed good CI outcome (Fig. 3, red, yellow and pink).

Altogether, our findings concur to suggest that maintaining language-related processes in the left posterior temporal cortex during deafness is important for good CI outcome^9^ and that lip-reading might be instrumental for this. Future proficient CI users might implicitly evoke audio-visual (lip-reading) speech representations during visual-based phonological access, and by doing so could preserve audio-visual phonological processes that are critical after implantation^2^. However, lip-reading did not directly predict post-CI speech scores in the current study. This might indicate that a good lip-reading level before deafness is not a sufficient natural protection against a shift of phonological processes to the right hemisphere and against a recycling of visual cortex in other linguistic processes. Lip-reading presumably needs to be actively maintained during deafness to preserve the cohesion of the left hemispheric speech network^44^. This speculation could have clinical relevance for subjects during post-lingual deafness, that is, training lip-reading prophylactically by dedicated cognitive programs could improve CI outcome. This hypothesis, however, requires specific testing.

Importantly, we show that some subjects decouple their visual cortex from left audio-speech processing when becoming deaf, and involve it in alternative language networks, through functional coupling with regions of the right temporal lobe; this may optimize the use of written material. When such reorganization happens, early CI outcome is limited presumably because the capacity of visual cortices to cooperate with hearing during the initial steps of auditory recovery is compromised (Fig. 4a). In a complementary way, Strelnikov *et al*.^6^ showed that the absence of interaction between the right occipital cortex and the right STS during an audio-visual speech task at an early post-CI stage is a good predictor of auditory recovery, since in this case the occipital cortex remains available for synergistic left audio-visual interactions (Fig. 4b). Whether left hemispheric dominance can progressively be restored after CI in patients who show a right-hemispheric reorganization is an important question. A longitudinal PET study showed that cross-modal plasticity in the right anterior temporal cortex can be reversed after implantation in deaf subjects^19^. Long lasting speech comprehension difficulties may hence result from persistent right hemisphere reorganization. Testing whether plasticity effects can effectively be reversed is the next challenge in this field of research^61^.

While previous results suggested that left occipito-temporal coupling underpins post-CI audio-visual synergy^6^, we provide here the complementary demonstration that right occipito-temporal coupling is detrimental to post-CI recovery (Fig. 4). Faster than average accurate phonological processing seems a good marker of right occipito-temporal reorganization in deaf adults, and could potentially constitute an easy-to-use predictor of poor CI outcome if confirmed on a larger cohort of CI candidates. From a more fundamental perspective, this study shows that the deaf brain faces a dilemma between efficiently adapting to deafness, and preserving a normal-like neural organization that maximizes the chances to revert to auditory communication.

## Methods

### Subjects

The study was approved of by the Inserm Ethics Committee (Protocol number C09-20) and performed in 35 native French speakers: 18 post-lingual profoundly deaf subjects, candidates for cochlear implantation (mean age ±s.d.=49 ±15 years), and 17 normal-hearing controls (mean age ±s.d.=42 ±13 years, with hearing thresholds≤25 dB HL on pure tone audiogram, 500–4,000 Hz), matched for gender and educational level. Demographic and clinical data of the deaf subjects are summarized in Supplementary Table 1. The etiologies and duration of deafness reflected the usual clinical diversity. All CI candidates, randomly selected from the CI candidates list, had comparable durations of severe-to-profound hearing loss (mean±s.d.=5±7 years), except S14 who became profoundly deaf during childhood, yet after language acquisition (7 years old). In all participants, hearing was assessed by routine pure tone threshold evaluation using headphones. In CI candidates, speech tests were performed in free field conditions with their hearing aids on. All subjects, but S3 who was suspected of auditory neuropathy, were fitted with hearing aids during the period before surgery (at least on one side). Eleven of them went to university, or studied after graduating (mean post-grad duration: 3 years, range 1–5). Five subjects stopped their studies after graduation, and 2 before the age of 12 years (S6 and 13). This unusually large proportion of subjects with post-graduate level was controlled for in the selection of the normal-hearing group, along with age. None of the 18 CI candidates used sign language; they all relied either on lip-reading or written language for communication, depending on their lip-reading level (Supplementary Table 1).

All 18 deaf subjects met the French criteria for receiving a CI (speech perception below 50% word recognition at 60 dB in free field, with best-fitted hearing aids). They received a CI after completing a series of behavioural tests. The side receiving the CI was chosen with the patient and according to the pre-implant residual pure tone hearing thresholds. The worse ear was generally implanted^1^ since the implanted side has no influence on CI scores in post-lingually deafened subjects^1^,^63^. The four brands were represented: 12 Cochlear devices, 3 Advanced Bionics devices, 2 Med-El devices, and 1 Oticon Medical device. A CT scan was systematically performed (routine examination in the centre) to check for correct position within the scala tympani and angle of insertion (range in our group: 257–496° depending on the electrode-array used). All subjects presented with full insertion of the electrodes; except for the most basal electrode in four cases, all electrodes were functional in the remaining subjects. Speech perception with the CI alone (no hearing aid on the non-implanted side) was evaluated 6 months after the first fitting in free field, with signal presented in front of the subject (regular testing set up in the centre). We determined the rate of correctly repeated French words played from a CD in free field at 60 dB SPL.

The control sample included 17 participants matched with the deaf patients with respect to age and educational level (over 18 controls, one normal-hearing subject had to be excluded due to a technical problem during the data acquisition). Eleven of the normal-hearing controls went to university, or were still studying (mean duration of university studies=4 years, range 2–5), while the 6 remaining controls stopped studying after graduation.

All 35 subjects performed behavioural experiment (phonological testing and its control task, see protocol below). Accuracy and reaction times were measured. Once implanted, the CI speech scores of the 18 deaf subjects were recorded as described above. Among the 18 CI candidates, deaf subjects S1 to S11 agreed to perform the tasks in the fMRI scanner. The 22 subjects who participated in the fMRI scanning (deaf subjects numbered 1 to 11, and 11 normal-hearing controls) had normal or corrected-to-normal vision, no history of neurological pathology. According to the Edinburgh handedness inventory index^64^, two deaf subjects and one control were left-handed. The 11 deaf subjects enroled in the fMRI experiment had more testing: lip-reading was assessed preoperatively by testing the percentage of correct repeated phonemes of monosyllabic words silently pronounced by a speech therapist during a recorded movie.

### Stimuli and experimental protocol

Subjects performed rhyme decisions on two written items presented simultaneously on a black screen. These items were pseudo-homophones, that is, misspelled French words (for example, ‘afrikenne' versus ‘meccicaine' instead of africaine (from Africa) versus mexicaine (from Mexico)). Decisions could not be based solely on orthography but required phonological analysis (internal auditory conversions^33^,^34^). Pairs were controlled for length and number of syllables. Sixty-six pairs of pseudo-homophones were created. The experimental protocol was as follows: a 2-s instruction was shown before presenting 5 consecutive pairs. The instruction was followed by a 2-s blank screen. Each pair was presented for 3 s, followed by a 3-s blank screen with a ±0.5-s temporal jitter. Subjects gave their answer by button-press, as quickly as possible after the appearance of the pair on the screen. Subjects were discouraged to read aloud.

The control task was based on word spelling. Pairs were randomly chosen. Subjects were asked to tell whether the final letters of the 2 items were the same, by button-press. Similar to the phonological task, a 2-s instruction followed by a 2-s blank screen, preceded the 5 following pairs. Each pair was presented for 3 s, followed by a 3-s blank screen with a ±0.5-s jitter. The screen presentation was exactly the same for both tasks (font, white letters over a black background, centering) and the instructions led to the same choices (Yes or No) in order to keep motor reply and button-press-related attention identical.

Conditions (rhyming and control tasks), preceded by their instructions, were pooled into runs and repeated twice per run: 1 run comprised 8 blocks (1 block=1 instruction and 5 pairs of the same category). A 15-s blank screen followed each block. The order of presentation within and across conditions and subjects was randomized using MATLAB. Performance (per cent correct) and related reaction times (that is, for accurate trials, in milliseconds ms) were measured. The same experimental material and design were used for behavioural data acquisition (in 35 subjects) and functional imaging (in 22 of them). Subjects were first trained with pairs that were not included in the final sample, but presented with the same block design as the rest of the experiment. The software and size of the screen were the same.

### fMRI parameters

Gradient echo-planar fMRI data with blood oxygenation level dependent (BOLD) contrast were acquired with a 3-T Siemens Trio TIM, using the standard 12-channel head coil. Subjects' head movements were restrained by additional padding, inside of the head coil. Functional images, covering the whole brain, were acquired using a BOLD sensitive gradient echo planar imaging (EPI), employing the following acquisition parameters: Slices, 45; matrix size, 96 × 96; pixel size, 2.1 × 2.1 × 2.9 mm^3^; echo time, 30 ms; repetition time, 2,800 ms. A high-resolution T1-weighted image was acquired at the end of the scanning (slices, 176; echo time, 4.18 ms; repetition time, 2,300 ms; flip angle, 9°; pixel size, 1 × 1 × 1 mm; matrix size, 256 × 256). Earplugs (mean sound attenuation of 30 dB) and earmuffs (mean sound attenuation of 20 dB) were provided both to controls and deaf subjects to equate experimental environment.

### Statistical analyses on behavioural data

Mean phonological performances and reaction times (RT) were compared between groups (deaf subjects versus controls) using T-tests (two-tailed). Results are indicated in means±s.d. Correlations across behavioural parameters were tested using Pearson correlations (two-tailed), and linear regressions were used to search for statistical relationships between the tested parameters.

### Functional images statistical analyses

The fMRI data were analysed using SPM5 (Statistical Parametric Mapping, Centre for Neuroimaging, London, UK, http: //www.fil.ion.ucl.ac.uk/spm) in a Matlab 7.1 (Mathworks, Natick, MA, USA) environment and displayed using MRIcron software (www.sph.sc.edu/comd/rorden/mricron). We performed standard preprocessing (realignment and unwarping, normalization and spatial smoothing with an 8-mm full width at half-maximum Gaussian kernel), and calculated contrast images versus baseline in each single subject for each condition (pseudo-homophone, and control task). Age at fMRI for both groups was entered as a covariate in the following analyses.

Within-group phonological analyses: Contrasts of phonological versus control tasks (Ct) were computed (one-sample *t*-tests FDR-corrected for multiple comparisons, *P*<0.05, Fig. 2a).

Between-group phonological analyses: An ANOVA (groups × conditions, *P*<0.05, FDR corrected) examined the main effects of the phonological condition in each group. From this contrast, individual beta values were extracted from peak voxels of significant clusters, and compared between groups (Fig. 2b).

Whole brain correlations with behavioural measurements in deaf subjects: We used contrast images from the pseudo-homophone condition (minus baseline) in a regression analysis to test whether neural activation varied as a function of reaction times, lip-reading ability before cochlear implantation, deafness duration and CI speech scores 6 months post-implantation. We considered significant effect associated with *P*<0.001, uncorrected (Fig. 3). The variables were purposely not orthogonalized against each other to be able to appreciate the spatial overlap across the different effects. Activity in fronto-parietal regions (see the results of the whole brain negative correlation with RT in the deaf group in Fig. 3) possibly related to motor orienting and attention/working memory was controlled for by the control task, as also shown by the results (surface rendering) of whole brain correlation with RT in the control group (Supplementary Fig. 2).

Of note, Subject 6 (Supplementary Table 1) had fast RTs but poor accuracy during the rhyming task (Fig. 1b), and poor speech comprehension scores at 6 months post CI. S6 represents a profile of CI candidate with poor general phonological abilities, and thus reflects the clinical variability observed in everyday practice. Another subject (S13) had a similar profile.

Comparison of functional connectivity between groups: from the previous results (ANOVA deaf>controls and correlations with reaction times), we identified regions that were more strongly activated in deaf subjects relative to controls and participated in the accelerated performances at the same time. These regions were used as seeds to explore the functional specificity of their interactions with the rest of the brain. We used PPI to assess, where functional coupling during the PH condition was enhanced relative to the control task. This method computes cross-regional correlations between residual BOLD fluctuations and psychological contexts that are not accounted for by the main task effects^42^. One sample t-tests per group and two sample t-tests between groups were performed independently for each seed region. Results associated with a *P*<0.001 uncorrected are displayed in Fig. 3 (as black arrows), and displayed in Supplementary Fig. 3. We finally tested for a relationship between the strength of the occipito-temporal coupling and CI outcome. The results are sketched in Fig. 4a.

### Data availability

The datasets generated during and/or analysed during the current study are available from the corresponding author (D.S.L.) on reasonable request.

## Additional information

**How to cite this article:** Lazard, D. S. & Giraud, A.-L. Faster phonological processing and right occipito-temporal coupling in deaf adults signal poor cochlear implant outcome. *Nat. Commun.* **8**, 14872 doi: 10.1038/ncomms14872 (2017).

**Publisher's note:** Springer Nature remains neutral with regard to jurisdictional claims in published maps and institutional affiliations.

## Supplementary Material

Supplementary Information Supplementary Figures and Supplementary Tables.
