# Supplementary material for: Faster phonological processing and right occipito-temporal coupling in deaf adults signal poor cochlear implant outcome
Source: Nat Commun. 2017 Mar 28;8:14872. doi: 10.1038/ncomms14872 (PMC5379061; doi:10.1038/ncomms14872)
Supplement: Supplementary Information — Supplementary Figures and Supplementary Tables. [file ncomms14872-s1.pdf]

Hemispheric dominance in post-lingual deaf adults; Lazard & Giraud

Supplementary Figure 1

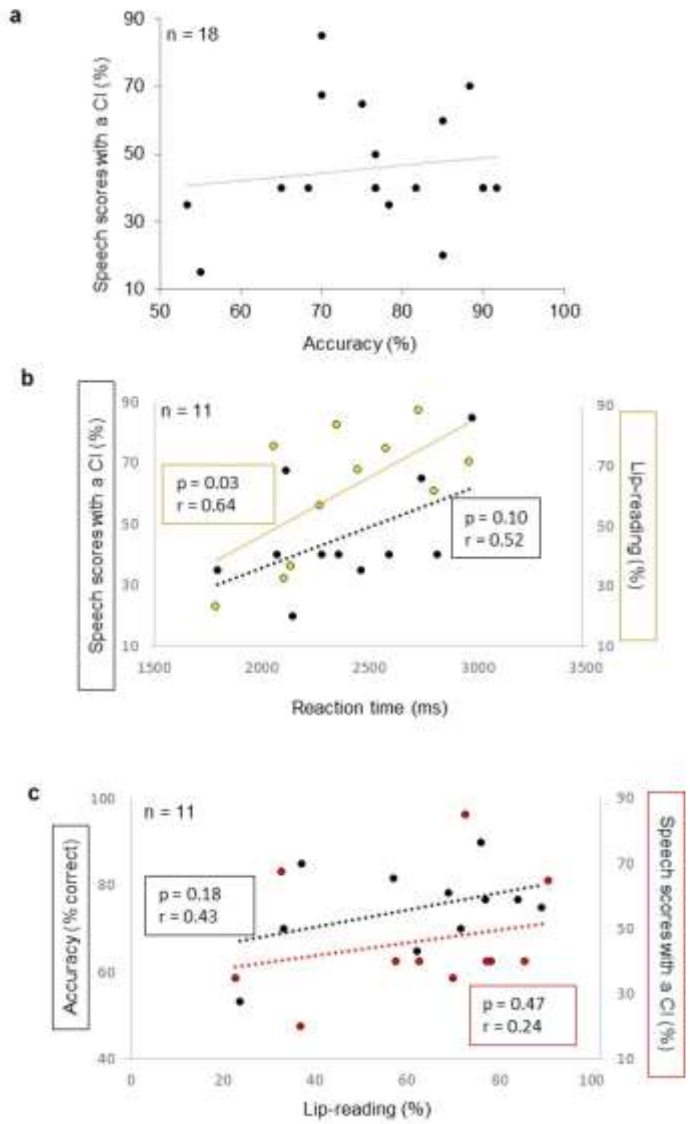

a. Post-cochlear implant (CI) scores (speech comprehension, % correctly repeated words) in post-lingual deaf subjects as a function of accuracy in the rhyming task (% correct). Accuracy did not predict CI outcome (no statistical correlation; the dotted line represent theoretical linear regression line). Note that two subjects obtained the same scores in the tests as two other subjects, and are consequently overlapping. b. Post-CI speech scores (in black) and lip-reading ability (% correct repeated phonemes, in yellow) as a function of RT in the 11 deaf subjects who performed the whole set of experiments. The predictive value of RT with respect to post-CI speech scores became significant when enlarging the group to 18 subjects (Figure 1d). A positive correlation was observed between lip-reading and RT. c. Performance during the rhyming task (in black) and CI speech scores (in red) as a function of lip-reading. There is no statistical relation between these factors.

Supplementary Figure 2

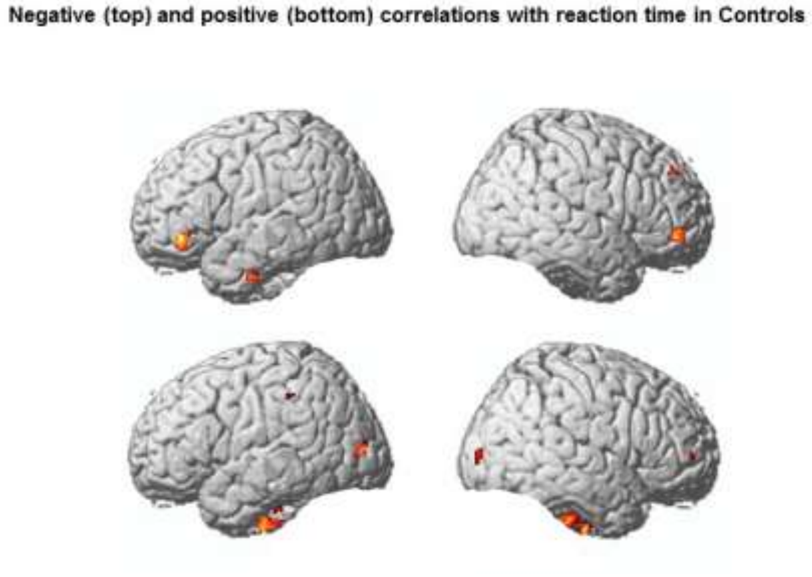

Surface rendering of correlations with RT during the phonological task in controls. Effects displayed on the figure are significant at $p \leq 0.001$, uncorrected. These results show that the effects obtained in the deaf group for the same correlation are specific to this sub-group and may not be related to button press, eye movements, or attention.

Supplementary Figure 3

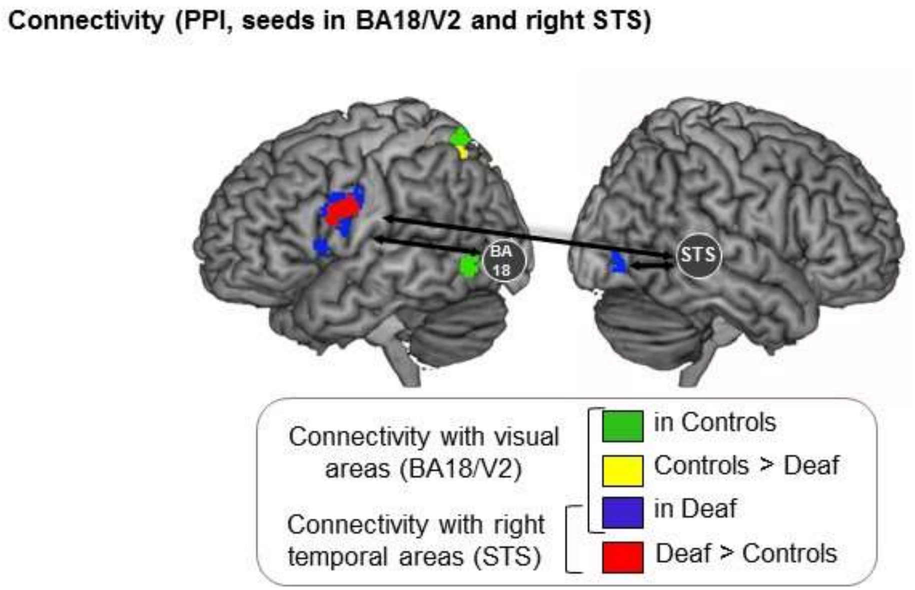

Connectivity analyses in the pseudo-homophone condition (relative to orthography). We used psychophysiological interactions that probed enhanced non-directional connectivity between the seed regions and the rest of the brain. The effects are displayed at $p<0.001$, uncorrected. For the seed region in Brodmann Area 18 (visual cortex), results are displayed in green for controls and blue for deaf subjects. Significant controls > deaf difference is shown in yellow. The contrast deaf subjects > controls was null. For the seed region in the right superior temporal sulcus (STS), results are displayed in blue for deaf

Supplementary Table 1

Clinical data for the 18 cochlear implant candidates. S1 to S11 were enrolled in the fMRI experiment

Subject number	Sex	Etiology	Age at fMRI (y)	Duration of s/p HL (y)	HA (Y/N)	Lip reading (phonemes, % correct)	WRS pre-CI* (%, 60 dB SPL)	WRS post-CI (%, 60 dB SPL)
1	F	Unknown	20.8	1	Y	76	0	40
2	M	Otosclerosis	58.0	0.5	Y	50	0	35
3	M	Unknown	41.9	5	N	79	15	65
4	M	Genetic	42.9	0.6	Y	71	0	85
5	F	Unknown	34.8	8	Y	80	0	40
6	M	Labyrinthitis	61.3	1	Y	29	0	35
7	M	Unknown	43.3	4	Y	78	5	67.5
8	M	Genetic	51.1	0.5	Y	33	0	40
9	F	Unknown	23.0	1.5	Y	74	15	40
10	F	Otosclerosis	60.2	1.5	Y	71	0	40
11	M	Unknown	59.6	0.5	Y	73	20	20
12	M	Ménière	67.0	5	Y	na	76	60
13	M	Unknown	78.7	5	Y	na	38	15
14	F	Unknown	55.2	31	Y	na	24	70

15	F	Chronic otitis	53.7	3	Y	na	0	50
16	F	Unknown	43.7	1	Y	na	0	40
17	F	Unknown	28.7	4	Y	na	29	40
18	F	Ototoxic	53.8	15	Y	na	0	40

Duration of severe to profound HL (s/p HL): time from the time point when the patient could no longer use hearing alone to communicate, even with the best-fitted hearing aids, and/or understand TV, and/or stopped using the telephone to the day of first fitting of the CI.

HA: hearing aid, Y: yes; N: no, na: not available, WRS: word recognition scores, * With optimally fitted hearing aids, Clinical French test with three-phoneme monosyllabic words.

Supplementary Table 2

ANOVA Deaf> Controls (Pseudohomophones > Control task)

**Brain region**	**MNI coordinates (x y z)**	**FDR corrected p value**	**n voxels**
Left visual cortex	-24 -88 4	0.001	146
Right visual cortex	26 -88 4	0.01	32
Left superior parietal cortex	-44 -46 58	0.005	66
Right posterior STS	56 -32 2	0.01	68

STS : superior temporal sulcus

Supplementary Table 3

Significant activation for multiple correlations with reaction times (RT), CI scores, lip reading ability (LR) and deafness duration in deaf subjects for the rhyming task

Brain region	MNI coordinates (x y z)	uncorrected p value	n voxels	Z score
**Negative corr. with RT**				
Left sup. frontal gyrus	-28 54 24	0.0001	62	4.04
Left inf. frontal gyrus	-52 20 14	0.0001	20	4.00
	-54 6 26	0.0001	29	3.65
Right inf. frontal gyrus	44 10 30	0.0001	31	3.86
Left sup. parietal lobe/postcentral gyrus	-36 -34 54	0.0001	104	4.08
Left visual cortex	-30 -94 -8	0.0001	15	3.62
Right visual cortex	38 -92 -4	0.0001	36	3.83
Right posterior STS	44 -22 -2	0.0001	43	3.94
**Positive corr. with RT**				
Left posterior STS/ STG	-56 -46 8	0.0001	19	3.81
**Positive corr. with CI scores**				
Left posterior STG	-42 -60 28	0.0001	38	3.68
**Negative corr. with CI scores**				
Left sup. frontal gyrus	-16 58 28	0.001	12	2.97

Left inf. frontal gyrus	-60 12 26	0.001	10	2.91
Left sup. parietal lobe/postcentral gyrus	-44 -30 48	0.0001	220	4.86
Right sup. parietal lobe	48 -38 62	0.0001	20	3.39
Left visual cortex	-32 -94 2	0.0001	27	3.95
Right posterior STS	46 -36 6	0.0001	21	3.91
**Positive corr. with LR ability**				
Left posterior STS/STG	-56 -46 6	0.001	10	2.98
**Negative corr. with LR ability**				
Left sup. parietal lobe/postcentral gyrus	-20 -56 68	0.0001	69	3.55
Right visual cortex	32 -90 -6	0.0001	106	4.22
**Negative corr. with deafness duration**				
Left sup. parietal lobe	-44 -62 56	0.0001	29	3.97
Left inf. temporal gryus	-56 -28 -22	0.0001	47	3.97

There was no positive correlation with deafness duration at that threshold.

Sup.: superior, inf.: inferior, STS: superior temporal sulcus, STG: superior temporal gyrus.
